# Polyamine Homeostasis in Development and Disease

**DOI:** 10.3390/medsci9020028

**Published:** 2021-05-13

**Authors:** Shima Nakanishi, John L. Cleveland

**Affiliations:** Department of Tumor Biology, H. Lee Moffitt Cancer Center & Research Institute, Tampa, FL 33612, USA; john.cleveland@moffitt.org

**Keywords:** polyamines, development, aging, metabolism, cancer

## Abstract

Polycationic polyamines are present in nearly all living organisms and are essential for mammalian cell growth and survival, and for development. These positively charged molecules are involved in a variety of essential biological processes, yet their underlying mechanisms of action are not fully understood. Several studies have shown both beneficial and detrimental effects of polyamines on human health. In cancer, polyamine metabolism is frequently dysregulated, and elevated polyamines have been shown to promote tumor growth and progression, suggesting that targeting polyamines is an attractive strategy for therapeutic intervention. In contrast, polyamines have also been shown to play critical roles in lifespan, cardiac health and in the development and function of the brain. Accordingly, a detailed understanding of mechanisms that control polyamine homeostasis in human health and disease is needed to develop safe and effective strategies for polyamine-targeted therapy.

## 1. Introduction

Polyamines are organic polycations that are found in all eukaryotes and nearly all prokaryotes. These molecules are essential for cell growth and survival [1,2,3,4] and affect various cell processes, where their primary and secondary protonated amino groups allow avid electrostatic interactions with negatively charged DNA, RNA, proteins, and phospholipids [5,6,7]. This property, and a series of molecular and functional studies, have shown that polyamines regulate chromatin organization, DNA replication, transcription, translation, ion transport and membrane dynamics.

**Figure 1:**
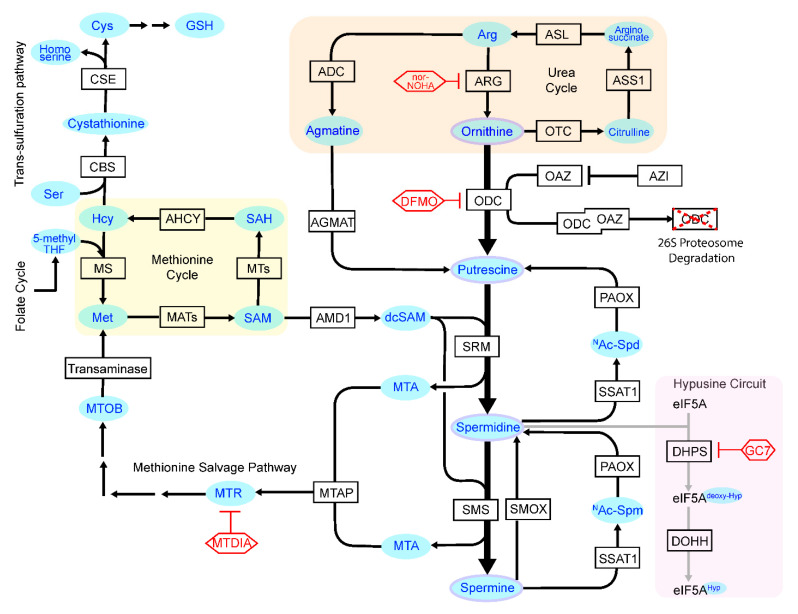
Polyamine metabolism and interacting pathways. Enzymes that control central polyamine biosynthesis and catabolism are shown, as well as the metabolic circuits that feed into the control of polyamine homeostasis. Light blue, substrates and products; red, inhibitors of key enzymes. ADC, Arginine decarboxylase; AHCY, S-adenosylhomocysteine hydrolase; AMD1, Adenosylmethionine decarboxylase-1; AGMAT, Agmatinase; Arg, Arginine; ARG, Arginase; ASL, Arginosuccinate lyase; ASS1, Arginosuccinate synthase-1; AZIN1, Antizyme inhibitor-1; Ac-Spd, *N^1^*-acetylated Spd; Ac-Spm: *N^1^*-acetylated Spm; CBS, Cystathione β-synthase; CSE, Cystathionine γ-lyase; Cys, Cysteine; DFMO, Difluoromethylornithine; DHPS, Deoxyhypusine synthase; DOHH, Deoxyhypusine hydroxylase; eIF5A, Eukaryotic translation initiation factor 5A; GC7, N1-guanyl-1, 7-diamine-heptane; GSH, Glutathione; HS, Homocysteine; Hyp, Hypusine; MATs, Methionine adenosyltransferases -1, -2A and -2B; Met, Methionine; MTA, 5′methylthioadenosine; MTAP, MTA phosphorylase; MTDIA, Methylthio-DaDMe-Immucillin-A; MTOB, 4-Methylthio-2-oxobutanoic acid; MTR: 5′ methylthioribose; MTs, Methyltransferase; MS, Methionine synthase; nor-NOHA, Nω-hydroxy-nor-arginine; ODC, Ornithine decarboxylase; OAZs, ODC antizyme-1, -2 and -3; OTC, Ornithine transcarbamylase; PAOX, Polyamine oxidase; SAH, *S*-adenosylhomocysteine; SAHH. SAH hydrolase; SAM, *S*-adenosylmethionine; Ser, Serine; SSAT1, SPD/SPM acetyltransferase 1 (SAT1); SMOX, Spermine oxidase; SMS, Spm synthase; SRS, Spd synthase.

Primary sources for polyamines in mammals are food intake, microbial synthesis in the gut and biosynthesis in cells. Polyamine biosynthesis is initiated by ornithine decarboxylase (ODC), which catalyzes the production of putrescine (PUT) via decarboxylation of ornithine; this is the rate-limiting step of the pathway (Figure 1). Further, another source of PUT is agmatine, where arginine-derived agmatine is converted by agmatinase (AGMAT) to PUT. This alternative pathway is well studied in lower organisms, yet there is limited information regarding its significance in mammals [8,9,10,11,12]. The higher order polyamines, spermidine (SPD) and spermine (SPM) are then generated from PUT by spermidine synthase (SRM) and spermine synthase (SMS), respectively, following the addition of aminopropyl groups from decarboxylated *S*-adenosyl-l-methionine (dcSAM) that is produced by adenosyl-l-methionine decarboxylase (AMD1) that decarboxylates SAM (Figure 1). Further, SPM can be oxidized via spermine oxidase (SMOX) to produce SPD, and *N*^1^-acetyl-SPD and *N*^1^-acetyl-SPM that are produced by spermidine/spermine *N*^1^-acetyltransferase (SSAT) are oxidized by *N*^1^-acetylpolyamine oxidase (PAOX) to recoup PUT and SPD, respectively.

Interestingly, polyamine homeostasis is maintained in a cell type and tissue-specific manner, where intracellular polyamine levels are tightly regulated by complex feedback mechanisms [13,14] that include: (i) transport; (ii) control of *ODC*, *AMD1*, and *SSAT1* transcription; (ii) polyamine-dependent control of *ODC* and *SSAT1* mRNA turnover; (iii) SPD-dependent control of translation initiation of ODC and SSAT; (iv) a polyamine-dependent +1 ribosomal frameshift that allows synthesis of a dedicated inhibitor of ODC coined antizyme (OAZ1) that directs proteasome destruction of ODC and controls polyamine transport [15]; (v) polyamine-dependent control of the transcription of an ODC decoy coined antizyme inhibitor (AZIN) that dampens antizyme activity [16,17]; (vi) feedback control of enzyme activity (e.g., induction of AMD1 activity following inhibition of ODC [18]); and (vii) polyamine control of ubiquitin-independent/NQO1-dependent proteasome destruction of ODC [19], which has one of the shortest half-lives of any protein in the cell [20]. Moreover, select enzymes in the polyamine biosynthesis pathway also respond to broad regulators of physiology, where there is circadian control of *Odc*, *Srm* and *Amd1* expression, and of polyamine levels, in a diurnal manner with peak levels during the night (~16 zeitgeber time), whereas polyamine catabolism appears relatively constant throughout day/night cycles [21].

Polyamine levels are normally maintained within narrow physiological ranges, and altered polyamine metabolism is sufficient to provoke various pathologies, including cancer, neurological defects and aging [22,23]. For example, elevated levels of polyamines and the polyamine biosynthetic enzymes ODC and AMD1 are often associated with hyper-proliferative phenotypes and are overexpressed in many cancer types. Indeed, forced overexpression of ODC in NIH 3T3 fibroblast cells is sufficient to induce tumors in immune compromised mice [24] and increased expression of ODC accelerates tumor development in premalignant epidermal cells [25]. In contrast, depletion of polyamines by knockout of *Odc* or treatment with an ODC suicide inhibitor coined α-difluoromethylornithine (DFMO) [26], markedly impairs tumor development in Eμ-*Myc* mice, a validated model of *MYC*-driven B cell lymphoma [27] and in MYCN-driven neuroblastoma [28,29]. Moreover, DFMO maintenance therapy can prevent relapse following completion of standard therapy and improves overall survival in pediatric patients with high-risk neuroblastoma (HRNB) [30]. In contrast, activation of polyamine catabolism appears to contribute to aging [31] and to tissue damage in response to injury, and is observed in various pathological conditions that result in cell damage [32,33]. Finally, overall levels of polyamines have been reported to decline with age [34], and polyamine supplementation, particularly of SPD, has been shown to extend lifespan in several model organisms, including budding yeast (*S. cerevisiae*), *Drosophila*, nematodes (*C. elegans*), human immune cells [35], and mice [36], suggesting beneficial effect of polyamine supplementation in human health.

The general functions of polyamines and polyamine feedback control mechanisms have been the subject of several reviews [3,13,14,37,38,39]. Here we review the most recent studies showing essential roles of polyamine homeostasis in human health and disease, and discuss the various mechanisms by which modulating polyamine levels can lead to both pathologies or benefits to health. Further, we discuss studies suggesting that the metabolic wiring of cancer cells makes them more sensitive to modulation of intracellular polyamine levels.

## 2. Beneficial Aspects of Polyamines and Effects of Polyamine Loss on Health

Polyamine levels decline with age in mice (e.g., 26-week old mice) [34], and recent studies assessing relationships between aging and polyamine levels in human liver tissues have revealed that the levels of PUT increase with aging, while the levels of SPM significantly decrease [40]. This observation is partly explained by age-associated increase in the expression of SMOX, and by decreases in the expression of ODC and AMD1 with aging. In contrast the levels of SPD do not correlate with age in humans and, unlike observations in mice, there are large variations in polyamines in humans that likely reflect individual-specific effects of differences in the diet and microbiome.

### 2.1. Spermidine and Spermine Can Extend Lifespan

Quite strikingly, supplementation of SPD has been shown to promote longevity in *S. cerevisiae*, nematodes, *Drosophila* and mice, and SPD also promotes the survival of human immune cells. At least in yeast this appears mechanistically to be due to direct inhibition of histone acetyltransferase (HAT) activity and to the induction of autophagy [35]. Indeed, dual knockout of IKI3, an essential subunit of the histone acetylating elongator complex, and of the HAT Sas3p (Δ*iki3*Δ*sas3*) phenocopies the effects of SPD treatment on lifespan in yeast, including reduced histone H3 acetylation, enhanced survival during chronological aging, and reduced ROS production and necrotic cell death. Further, depletion of polyamines by deletion of the yeast *ODC* gene, *SPE1* (Δ*spe1*) shortens the lifespan of yeast, and this is ameliorated, in part, by dual loss of IKI3 and Sas3p (Δ*spe1*Δ*iki3*Δ*sas3*). Thus, in yeast, aging-related changes in HAT activity acts downstream of SPD. 

The effects of SPD on HAT activity lead to global hypoacetylation, a scenario that should suppress gene transcription. However, expression profiling studies have rather revealed that several genes encoding components of the autophagy pathway are up-regulated by SPD treatment, and the most significant of these is Atg7, which functions as an essential E1 enzyme that is necessary for the induction of the pathway. This has been speculated to be due to the induction of a fail-safe compensatory mechanism that ensures the activation of this recycling center that provides essential building blocks necessary for cell survival. In accord with this notion, loss of ATG7 in yeast or flies, or depletion of BECLIN-1 (Atg6) in *C. elegans*, abolishes SPD-induced lifespan extension; thus, autophagy is essential for SPD-induced longevity [35] in these higher organisms.

Lastly, as highlighted in a recent article by Holbert et al. [41], one caveat of mammalian cell culture based studies evaluating the biological effects of SPD supplementation is the fact that SPD and SPM are rapidly oxidized by amine oxidase that is present in, for example, bovine serum, and thus looking at selective effects of SPD supplementation should be done (where feasible) in the presence of aminoguanidine, an effective inhibitor of amine oxidase. That is, the effects on SPD supplementation on the induction of autophagy could be due, at least in part, to oxidation of polyamines [41].

### 2.2. Cardioprotective Effects of Spermidine

Interestingly, increased lifespan in mice that is induced by oral uptake of SPD delays cardiac aging by reducing hypertrophy and improving diastolic function. Further, in accord with findings on SPD-induced longevity via the induction of autophagy [35], SPD supplementation increases autophagic flux in aging cardiomyocytes in vivo, but cannot rescue systolic dysfunction, and age-related cardiac deterioration is manifest in mice that selectively lack Atg5 in cardiomyocytes (*Atg5*^fl/fl^;*MLC2a*-*Cre*^+^ mice) [ 36]. Finally, in humans, the dietary intake of SPD, which has been estimated on the basis of diet surveys, inversely correlates with risk of cardiovascular disease [36].

### 2.3. Neuroprotective Effects of Polyamines

PUT, SPD, and SPM are found throughout the human brain, but are uniquely distributed in both a temporal and spatial manner, suggesting that each polyamine has specific roles in brain development, homeostasis and/or function. For example, levels of SPD increase drastically after birth, and reach maximum levels at ~40 years of age [42].

Links between polyamines and ageing-associated decline of cognitive function such as learning and memory have also been documented. Indeed, the levels of both SPD and PUT decrease in the brains of aging flies, concomitant with decline of olfactory memory, and restoration of polyamine levels by dietary SPD supplementation is sufficient to suppress age-induced memory impairment (AMI) [43] and maintain synaptic flexibility [44,45]. Importantly, such SPD-mediated protection from AMI does not appear to be a result of increased lifespan or generally improved health, but is rather due to intrinsic effects on regulating olfactory memory in aging flies. Moreover, specific overexpression of *Odc* in Kenyon cells, which are neurons that comprise the mushroom body of *Drosophila* brains and which are important for olfactory memory formation, is sufficient to suppress AMI. Finally, SPD supplementation fails to prevent memory impairment in *Atg7^−/−^* and *Atg8a^−/−^ Drosophila*, suggesting autophagy is required for SPD-mediated protection from AMI. Furthermore, recently, Madeo’s group showed that SPD supplementation improves cognitive performance in flies and aged mice, which appears to be due to the effects of SPD on enhancing mitochondrial function in neuronal tissue through autophagy and mitophagy [46]. Additionally, a large-scale food survey studies revealed that higher dietary SPD intake is correlated with a reduced risk for age-related cognitive impairment and decline in humans [46].

Loss of SPM due to mutations in spermine synthase (SMS) is known to cause Snyder-Robinson syndrome (SRS), an-X-linked intellectual disability [47] that includes other symptoms, in particular hypotonia, skeletal defects, movement disorders, speech/vision impairment, seizure and cerebellar dysfunction [48]. Along with SPM, the levels of PUT are significantly reduced in SRS patient-derived lymphoblast cell lines versus wild type lymphoblast cells lines, whereas SPD levels are increased. Thus, SPD/SPM ratios are elevated in SRS patient cells, and uptake of exogenous spermine via polyamine transport in SRS cells can restore proper ratios of SPD/SPM [49]. Furthermore, in *Drosophila* SMS (dSMS) is ubiquitously expressed throughout the nervous system and homozygous *SMS*-deficient flies that are incapable of converting SPD to SPM exhibit retinal and synaptic degradation, consistent with the reports showing retinal pigmentary changes in SRS patients [50], and these phenotypes can be rescued by re-expression of wild-type dSMS [51]. Moreover, consistent with polyamine alterations seen in SRS patients, dSMS deficiency results in reduced levels of PUT and increases in SPD and SPD catabolism. In turn, this leads to the generation of reactive aldehydes and hydrogen peroxide that induce lysosomal dysfunction and oxidative stress, blocking autophagic flux, endocytosis and mitochondrial functions. Notably, excess reactive oxygen species (ROS) that accumulate because of excessive SPD catabolism in SMS deficiency can be mitigated by antioxidants that can at least partially restore mitochondria functions [51], suggesting such a strategy can be applied to therapeutically treat SRS patients.

Interestingly, interactions between the nervous and immune systems modulate intestinal homeostasis and polyamines play important roles in this process. Specifically, crosstalk between intestinal neuronal cells and muscularis macrophages limits neuronal damage following luminal infections [52], and this involves upregulation of neuroprotective β2-adrenergic receptor signaling and the arginase-1 (ARG1)-polyamine axis. Furthermore, supplementation with SPM, which is known to inhibit the inflammasome [53], rescues neuronal cell death provoked by infection of mice with *Salmonella typhimurium*, whereas mice treated with DFMO show increased loss of neuronal cells following such infections [52].

## 3. Detrimental Aspects of Polyamines: Effects of High Polyamine Levels

Intracellular polyamine concentrations are maintained through biosynthesis, catabolism and uptake/export. The enzymes/transporters controlling intracellular polyamine pools are tightly regulated at many levels including transcription, translation and degradation, and each has unique feedback control; i.e., each level of control responds to perturbations in polyamine pools. Accordingly, dysregulation of these enzymes can have severe consequences on human health, including promoting cancer.

### 3.1. Control of ODC

ODC is the rate limiting enzyme of polyamine biosynthesis and ODC levels are tightly controlled and are promptly adjusted to cellular needs by several mechanisms, including transcription, mRNA stability, translation and degradation [54]. *ODC* is a direct transcription target of MYC oncoproteins and is overexpressed in *MYC*- and *MYCN*-driven cancers [27,28,29,55]. The MYC-ODC-polyamine axis has been well studied and reviewed by others [56,57]. However, other aberrant signaling pathways also control ODC and polyamines in cancer. For example, binding of HuR, an AU-rich RNA binding protein, to AU-rich elements in the 3′ untranslated region (UTR) of *ODC* transcripts stabilizes *ODC* mRNA in models of skin cancer and in KRas-transformed cells, and this is partly dependent of mTORC1 signaling, as treatment with the mTORC1 inhibitor rapamycin, or siRNA targeting *mTOR*, destabilizes *ODC* mRNA by impairing HuR binding [58].

Polyamine dependent translation control of ODC is also important, where ODC translation is impaired by translation of an upstream open reading frame (uORF) [59]. Additionally, when cap-dependent ODC translation is blocked, ODC is translated from an internal ribosome entry site (IRES), and this level of control is manifest during mitosis [60]. Furthermore, alterations in the Sonic Hedgehog (Shh) pathway, which are a hallmark of medulloblastoma, promotes polyamine biosynthesis in cerebellar granule cell progenitors (GCPs) by engaging a non-canonical pathway that augments ODC translation. Specifically, a complex of the cytoplasmic Hedgehog (Hh) transducer Sufu with the small zinc finger protein CNBP promotes IRES-dependent translation of ODC [61]. Interestingly, AMPK-directed phosphorylation of CNBP is required for this process, indicating an oncogenic role of AMPK signaling in this context. Importantly, targeting this Hedgehog/AMPK-mediated ODC translational control blocks the growth of medulloblastoma cells ex vivo and in vivo by reducing polyamine levels [62]. Finally, ODC translation is impaired by the tumor suppressor p53 via effects on ammonium metabolism. Specifically, p53 suppresses the transcription of the *CPS1*, *OTC* and *ARG1* genes that drive the urea cycle, leading to increased ammonia levels that suppress ODC translation, but not its transcription [63].

Turnover of ODC protein levels is also tightly regulated. Indeed, ODC is a very short-lived protein in eukaryotic cells and, unlike many other proteins, ODC degradation via the 26S proteasome is independent of ubiquitination. Rather, as noted above, ODC destruction is orchestrated by binding of antizyme (OAZ1, 2, and 3) that directs ODC destruction by the proteasome [15,64]. In turn, antizyme function is harnessed by the ODC decoys AZIN1 and AZIN2 [16,17,65]. Importantly, translation of both OAZ and AZIN family members is regulated by polyamines, where high polyamine concentrations promote OAZ translation via +1 frameshifting on *OAZ* mRNA [15,66,67,68], while AZIN translation is repressed at high polyamine concentrations, via translation of a non-AUG-initiated upstream conserved coding region (uCC) [69]. While interaction of antizyme with the *N*-terminus of ODC is essential, the 37-residue *C*-terminus of ODC is critical for antizyme-mediated proteasome degradation [70].

The first case of a pediatric patient carrying a gain-of-function mutation in *ODC1* was recently reported as a new developmental disorder that includes global developmental delays, alopecia, overgrowth and dysmorphic features (Bachmann-Bupp syndrome) [71]. Strikingly, this heterozygous de novo nonsense mutation (c.1342 A > T) results in premature translation termination, generating a *C*-terminally 14-residue truncated ODC protein that is functionally active, and that is more resistant to proteasomal degradation. This leads to the accumulation of PUT in cells [71,72] and DFMO treatment, which has been safely used for decades in the treatment of African trypanosomiasis and hirsutism and which is now in clinical trials as a chemopreventative (e.g., prostate and colon cancer) or therapeutic (e.g., neuroblastoma) agent for several malignancies, may provide a therapeutic strategy for treatment of patients with this gain-of-function ODC1 variant.

There are only two proteins that are known to physically interact with ODC; the aforementioned OAZ and Sepiapterin reductase (SPR), an enzyme that mediates production of tetrahydrobiopterin (BH_4_), an essential cofactor of nitric oxide synthase (NOS). Interestingly, depletion of SPR by siRNA significantly impairs ODC enzyme activity and depletion or pharmacological inhibition of SPR impairs proliferation of neuroblastoma cells suggesting SPR as a potential new regulator of ODC [73,74]. Although the roles of the interaction between ODC and SPR remain largely unknown, it is also interesting that combination treatment of the SPR inhibitor sulfasalazine with DFMO shows synergistic antiproliferative effects versus neuroblastoma cells, at least ex vivo [74].

### 3.2. Regulation of AMD1

AMD1, which directs production of the aminopropyl donor necessary for the generation of SPD and SPM from PUT and SPD, respectively (Figure 1) [75], is also a transcription target induced by MYC oncoproteins [27]. Furthermore, AMD1 translation is also highly regulated. First, high levels of polyamines impair AMD1 translation by promoting ribosome pausing at an upstream open reading frame (uORF) in *AMD1* mRNA, thereby inhibiting translation initiation [76]. Second, recent ribosome profiling studies have shown that an accumulating queue of ribosomes that stall at a downstream stop codon near the 3′ end of *AMD1* mRNA, due to ribosome read-though, halt AMD1 translation, thus limiting the number of AMD1 molecules that can be synthesized from a single *AMD1* transcript [77].

AMD1 protein levels are also controlled by mTORC1-dependent regulation of proAMD1 stability via phosphorylation of S298 in the pro-enzyme. Specifically, metabolomic analyses of tumors from a *Pten*-deficient prostate cancer (PCa) mouse model, and of human PCa biopsies, revealed increases in dcSAM, *N*-acetyl-SPD and *N*-acetyl-SPM [78], and these changes were linked to increased levels of AMD1 protein but not mRNA. Further, re-expression of wild type but not catalytically inactive *PTEN* in *PTEN*-deficient PCa cells reduced AMD1 protein levels. Finally, elevated levels of AMD1 and its product dcSAM are manifest in PCa having activated mTORC1, and treatment with mTORC1 inhibitors provokes reductions in half-life of proAMD1 and this response can be reversed by proteasome inhibitors. Thus, a PI3K-PTEN-mTORC1 pathway promotes AMD1 protein stability and polyamine biosynthesis in prostate cancer.

Finally, the methionine salvage pathway can also affect AMD1 and polyamine levels in cancer. Specifically, the *MTAP* gene encoding 5-methylthioadenosine phosphorylase (MTAP), a key enzyme in the methionine salvage pathway that converts a by-product of polyamine synthesis, 5-methylthioadenosine (MTA), back to methionine, is frequently co-deleted with the tumor suppressor gene *CDKN2A/B* located on chromosome 9p21 [79], for example in Glioblastoma multiforme (GBM).

### 3.3. Control of Polyamine Catabolism and Transport

Homeostasis of polyamine content is also highly regulated through catabolism and transport. Here, *N*^1^-acetyl-SPD and *N*^1^-acetyl-SPM produced by SSAT (Figure 1) promotes polyamine export, and drives their catabolism via PAOX and SMOX, which can recoup levels of SPD and PUT, but which also generate cytotoxic metabolite byproducts, specifically hydrogen peroxide and reactive aldehydes. Thus, polyamine metabolism can be disrupted by targeting the catabolism of higher polyamines and dysregulation of polyamine catabolism leads to many pathological consequences.

SSAT is regulated at numerous levels. First, *SSAT* transcription is controlled by a conserved polyamine-responsive element (PRE) present in the 5′ regulatory region of the gene [80], and *SSAT* transcription is induced by a heterodimer comprised of Nrf-2 (nuclear factor erythroid 2-related factor 2) that is constitutively bound to the *SSAT* promoter, in complex with a cofactor coined polyamine-modulated factor-1 (PMF-1) [81,82]. Second, SSAT translation is markedly increased by treatment with polyamines or polyamine analogs [83], and this appears to occur by relieving translational repression that is mediated by an upstream open reading frame (uORF) [84]. Finally, SSAT translation is also suppressed by the interaction of a stem-loop structure near the translation initiation site of *SSAT* mRNA with nucleolin, and this is alleviated through autocatalysis of nucleolin that is provoked by high levels of polyamines [85].

SSAT was initially thought a particularly attractive target because it is highly inducible enzyme. Indeed, superinduction of SSAT by treatment with polyamine analog *N^1^*, *N^11^*-bisethylnorspermine (BENSpm) has showed some antitumor activity via H_2_O_2_ production, and depletion of naturally occurring polyamines [86,87]. Further, BENspm and a related analog coined DENspm have been tested in clinical trials, yet the greatest effect reported was only stabilization of disease [88,89,90,91,92].

Recently, one of the tumor-suppressive roles of p53 was linked to SSAT-induced cell death. SSAT is one of many p53 transcription targets that is induced by p53 activation upon DNA damage [93], and upregulation of SSAT induces lipid peroxidation and an iron-dependent form of cell death coined ferroptosis following ROS stress. Finally, loss of SSAT abrogates this form of cell death. Although the detailed mechanism is unknown, SSAT expression is correlated with arachidonate 15-lipoxygenase (ALOX15), a lipooxygenase that is critical for stress-induced cell death, and SSAT-induced ferroptosis is completely inhibited by ALOX15 inhibitor.

SMOX, FAD-dependent enzyme, directly back converts SPM to SPD and also generates toxic byproducts, H_2_O_2_ and 3-aminopropanol (3-AP). SMOX expression has been shown to be induced by a variety of inflammatory stimuli, and chronic oxidation of increased SPM has been linked to a number of pathologies including cancers through altered polyamine homeostasis and H_2_O_2_ accumulation. Upregulation of SMOX has also been reported in several diseases including inflammatory bowel diseases (IBD) (e.g., in colitis induced by Enterotoxigenic *B. fragilis*) [94], gastric inflammation and carcinogenesis that are manifest following infection by *Helicobacter pylori* [95], prostate carcinogenesis [96], and in chronic hepatitis and hepatocellular carcinoma [97].

It has been suggested that increased ROS production seen following polyamine catabolism activation evokes oxidative stress (e.g., DNA damage) that can trigger carcinogenesis or disease progression. Interestingly, when gastric epithelial cells from populations in the region of high gastric cancer risk in Columbia were cultured with *H. pylori*, greater levels of SMOX expression and DNA damage were observed compared with the samples from the low-risk region [98]. Mechanistically, the tumor suppressor miR-124 negatively regulates *SMOX* expression by binding 3′UTR of SMOX mRNA [99]. However, in gastric adenocarcinoma, miR-124 is highly methylated and thus, silenced, which allows upregulation of SMOX. In accord with this, miR-124 DNA methylation is significantly elevated in the gastric mucosa of the high-risk Columbian populations [99]. Finally, *Smox*^−/−^ mice infected with *H. pylori* have significantly reduced levels of gastric SPD and *H. pylori* induced inflammation, and reduced DNA damage and β-catenin activation [100].

Finally, the oxidation byproduct 3-AP spontaneously converts to acrolein, which is more toxic than H_2_O_2_ [101]. Notably, the levels of both oxidases, SMOX and PAOX, and acrolein are increased in plasma of brain stroke patients and the multiplied values of those three highly correlate with stroke severity [102]. In addition to brain infarction, acrolein also causes tissue damage during dementia, renal failure, and in primary Sjogren’s syndrome, possibly through conjugation and modulation of key proteins. Mechanistically, recent studies have shown that acrolein conjugation with tubulins inhibits dendritic spine extension [103], and that acrolein conjugation with vimentin and actin alters the structure of the cytoskeleton following brain infarction [104]. Importantly, levels of protein-conjugated acrolein are a biomarker for risk and severity of these diseases [105].

Although acrolein is known primarily as a cytotoxic byproduct, it is noteworthy that acrolein also plays essential physiological roles in bile canalicular lumen formation in liver cells [106]. Specifically, SMOX expressed in cells lining the bile canalicular lumen, which generates acrolein that alkylates and inactivates PTEN, allowing activation of AKT that stimulates F-actin remodeling for canalicular lumen formation. In accord with this funcion, treatment with acrolein scavengers, N-acetylcysteine (NAC) and *N*-benzylhydroxylamine (N-BHA) significantly reduces bile canalicular lumen formation [106].

Although polyamines have been shown to have neuroprotective effects (see above), it is also noteworthy that polyamine toxicity is implicated in neurodegenerative pathogenesis, including Kufor-Rakeb syndrome and early onset Parkinson’s disease. Further, a decrease in SSAT expression, which lead to increased levels of higher order polyamines, has been linked to α-synuclein aggregation, and to Parkinson’s disease [107]. Moreover, defects in lysosomal polyamine export have been recently implicated in neurodegenerative disorders [108]. Specifically, a newly identified lysosomal polyamine transporter, the P-type ATPase ATP13A2 (PARK9), promotes lysosomal efflux of SPM into the cytosol, and loss-of-function mutations in *ATP13A2* disrupts SPM-induced ATP13A2 efflux, which induces lysosome rupture and cell death.

High levels of polyamines in brain are also associated with other neurological disorders such as epilepsy. In particular, Cervelli and colleagues have shown that SMOX activity is linked to excitotoxicity using SMOX overexpressing (Dach-SMOX) mouse [109]. These mice are more sensitive to excitotoxic insult such as kainic acid (KA) treatment in comparison to control mice, with increased ROS, seizures, neurodegeneration and astrogliosis. Mechanistically, these effects are linked to protein kinase C (PKC) and Nrf2 activation, which in turn induces *SSAT* in astrocytes via its PRE and the Xc-transporter (cystine/glutamate antiporter) via antioxidant response element (ARE) [110], provoking glutamate release and excitotoxic stress [111,112,113].

### 3.4. Control of Polyamine Homeostasis and Interacting Metabolic Pathways in Cancer

Cancer cells undergo metabolic reprogramming, making them particularly sensitive to perturbation of select metabolic circuits. Indeed, recent studies have shown that altering polyamine metabolism adds strain (metabolic stress) on these circuits, and that, conversely, targeting metabolic circuits can affect polyamine homeostasis (Table 1). Accordingly, exploiting vulnerabilities of polyamine metabolic networks is an attractive therapeutic strategy to disable tumor cell metabolism.

Prostate cancer (PCa) is known to have high degree of polyamine metabolic flux, and therefore PCa is highly sensitive to changes in polyamine levels. For example, knockdown or inhibition of MTAP (S-methyl-5′-thioadenosine phosphorylase) by shRNA or treatment with Methylthio-DADMe-Immucillin-A (MTDIA) blocks androgen sensitive prostate cancer growth in vivo, suggesting this methionine salvage pathway is important for PCa cell survival [114]. Furthermore, activation of polyamine catabolism by the polyamine analog BENSpm and concomitant inhibition of MTAP by MTDIA synergistically block cell proliferation and provoke cell death in PCa cells ex vivo and in vivo [115]. In this scenario, BENSpm augments SSAT enzyme activity to produce acetylated polyamines, promotes export of acetylated polyamines [116,117], and also leads to depletion of intracellular polyamines [118], increasing demand for polyamine synthesis. This is then derailed by treatment with MTDIA, which inhibits methionine salvage pathway and cuts off the supply of SAM that is converted to dcSAM by AMD1 and is needed to provide amino-propyl groups to sustain polyamine synthesis (Figure 1). Thus, Enhancing polyamine flux can also be leveraged as a therapeutic strategy. Indeed, treatment with BENspm may have therapeutic potential in *MTAP*-deleted cancers with high degree of polyamine metabolic flux. 

In addition to polyamine synthesis, methionine is also an upstream precursor for cysteine synthesis (Figure 1). Cysteine is a non-essential amino acid, yet serves critical roles as a precursor for anabolic and antioxidant pathways (e.g., glutathione synthesis) that promote cell survival and proliferation and that are in high demand in cancer cells. Further, blocking extracellular sources of cysteine can lead to ferroptosis. Interestingly, a recent study by Zhang and colleagues [119] showed that there was a strong correlation between efflux of the polyamine synthesis byproduct MTA and sensitivity to cysteine starvation. In fact, inhibition of polyamine biosynthesis by treatment with an AMD1 inhibitor (sardomozide) or with MTOB (4-methyylthio-2-oxobutanoic acid), an intermediate in the methionine salvage pathway [120,121] (Figure 1), rescues cysteine-dependent cells from cysteine starvation [119]. In contrast, polyamine supplementation increase ROS levels and sensitizes these cells to cysteine starvation, and these effects are blocked by inhibition of SMOX and PAOX. Finally, *MTAP*-deleted cells exhibited strong sensitivity to cysteine starvation where loss of MTAP leads to increased AMD1 expression, polyamine levels, ROS and cell death [119].

It is also important to note that stress that is generated by metabolic perturbations are often context dependent. In the aforementioned study of Affronti et al. [115], where PCa cells have intrinsically high polyamine metabolic flux and rely on the methionine salvage pathway, pharmacological inhibition of the methionine salvage pathway depletes SAM pools and suppresses polyamine synthesis. In contrast, in other tumor cell types defects in the methionine salvage pathway augments polyamine levels [119].

The urea cycle is upstream of polyamine biosynthesis and, accordingly, alterations in this cycle affect polyamine homeostasis and are associated with cancer. In the urea cycle free ammonia is converted to carbamoyl phosphate in hepatocytes, which is then be converted to cytosolic arginine by arginosuccinate lyase (ASL) and arginosuccinate synthase 1 (ASS1). In turn, arginine can be converted to urea and to ornithine by arginase-1 (ARG1) or arginase-2 (ARG2) (Figure 1). Further, arginine-derived ornithine can be converted to: (i) glutamate-gamma-semialdehyde, a substrate for glutamate and proline synthesis by ornithine aminotransferase (OAT); or (ii) PUT by ODC. Finally, both OAT and ODC require the essential cofactor pyridoxal phosphate (PLP) which is an essential cofactor for approximately 4% of all cellular enzymatic reactions [122].

Reduced expression of key urea cycle enzymes including ASS1, ASL and ARG2 has been reported in a number of cancer types [123,124,125,126,127], suggesting tumor suppressive roles of these urea cycle enzymes. For example, loss or reduced expression of *ASS1* is manifest in several cancer types and ASS1-deficient cancer cells are totally reliant on extracellular arginine. Accordingly, arginine deprivation using pegylated arginine deiminase (ADI-PEG20), which degrades arginine to citrulline and ammonia, has shown promising initial results in the clinical trials [128] even though resistance to ADI-PEG20 has also been reported [129]. Interestingly, *ASS1* loss is associated with compensatory increases in the expression of *ODC1* and *AMD1*, and with reduced polyamine catabolism [130]. Further, ASS1-deficient cells have increased sensitivity to DFMO treatment versus ASS1-replete cells, suggesting a synthetic lethal interaction between *ASS1* loss and inhibiting polyamine synthesis. Indeed, DFMO plus ADI-PEG20 combination treatment has synergistic effects, which could be exploited for treatment of ASS1-deficient cancers.

Alterations in ARG2 have also been implicated in cancer. Specifically, ARG2 is frequently suppressed along with ASS1 and ASL in clear cell renal carcinoma (ccRCC), the most common subtype of kidney cancer [127,131]. Copy number loss of *ARG2* and *ASS1* genes are high in ccRCC patients (38% and 24%, respectively) and their combined loss connotes a nearly 3-year reduction in overall survival. Further, restoration of ARG2 expression suppresses ccRCC growth and this occurs via two mechanisms: (i) the depletion of PLP; and (ii) increased toxicity from the accumulation of polyamines [131]. Moreover, supplementing exogenous polyamines impairs the growth of ARG2-deficient kidney HK-2 cells.

Conversely, hyperactive arginine metabolism and excessive polyamine production have been observed in psoriasis, an incurable chronic inflammatory disease [132]. Specifically, deficiency in protein phosphatase 6 (PP6) disables skin homeostasis and this has been shown to involve increased *ARG1* transcription via phosphorylation and activation of the C/EBP-β transcription factor, and increased production of polyamines. Here increases in positively charged polyamines, which avidly bind to nucleic acids, is thought to facilitate self-RNA internalization by psoriatic keratinocytes and self-RNA sensing by myeloid dendritic cells. Notably, restoring the urea cycle using the nor-NOHA arginase inhibitor or treatment with DFMO has been shown to alleviate this inflammation, suggesting a new strategy for treatment of psoriasis.

**Table 1:** Polyamine metabolic vulnerabilities in cancer and other diseases.

Interacting Metabolic Pathway	Deregulated Enzymes/Genes	Target (Inhibitor)	Target Polyamine (Inhibitor/Compound)	Description	Disease	Ref.
Arginine pathway	*ASS1*	Arg depletion (ADI-PEG20)	ODC inhibition (DFMO)	ASS1-deficient cells have decreased levels of acetylated polyamines along with compensatory increases in polyamine biosynthetic enzymes.	Malignant pleural mesothelioma (MPM)	[130]
Arginine pathway/Urea cycle	*ARG2*	N/A	Polyamine toxicity	ARG2 suppresses tumor growth via depletion of biosynthetic cofactor PLP and toxic polyamine accumulation.	Clear cell renal cell carcinoma (ccRCC)	[131]
Urea cycle	p53 repressive target genes; *CPS1, OTC and ARG1*	N/A	ODC translation	p53-induced ammonia accumulation represses ODC translation.	Colon cancer	[63]
Arginine pathway/Urea cycle	*ARG1*	Inhibition of arginase (nor-NOHA)	ODC (DFMO)	Increased polyamine production in PP6-deficient keratinocytes facilitates self-RNA sensing by dendritic cells in psoriasis.	Psoriasis	[132]
Cysteine metabolism		Cysteine starvation	MTAP deletion	*MTAP* deletion upregulates polyamine pathway, which promotes ferroptosis under cysteine starvation.	Colorectal, breast and pancreatic cancers, and glioblastoma	[119]
Methionine salvage pathway		MTAP inhibition (MTDIA)	SSAT activation (BENSpm)	While keeping the high polyamines flux, SAM pools are depleted by inhibition of methionine salvage pathway.	Prostate cancers	[115]

## 4. Concluding Remarks

Intracellular polyamine pools are tightly regulated by complex mechanisms that maintain physiological levels of polyamines, and these are rapidly modulated in scenarios where there is a requirement for increased or decreased polyamines. As described above, strict control of polyamine levels is necessary as chronic imbalance of polyamine pools leads to the many detrimental consequences described above. Indeed, under such scenarios polyamines and polyamine metabolites can serve as diagnostic biomarkers. For example, as noted above, one of most toxic polyamine metabolites, acrolein, is used as a biomarker for stroke and renal failure [23].

Given the fact that both decreased and elevated polyamines result in unique pathological conditions, this makes targeting polyamines extremely challenging. For example, while SPD supplementation can delay aging in several organisms via the activation of autophagy, increased polyamines can also promote progression of different cancer types by driving cell proliferation. Thus, prolonged intake of dietary polyamines could potentially increase risk for some cancer types. Rigorous analyses of such unintended consequences, which could also include metabolic aberrations, should be a focus of future investigations.

## Data Availability

Not applicable.
